# Comparative analysis of IGFBP-3 gene sequence in Egyptian sheep, cattle, and buffalo

**DOI:** 10.1186/s13104-019-4657-6

**Published:** 2019-09-23

**Authors:** Ahmed. A. Saleh, Amr M. A. Rashad, Nada. N. A. M. Hassanine, Mahmoud A. Sharaby, Yongju Zhao

**Affiliations:** 1 College of Animal Science and Technology, Southwest University, Chongqing Key Laboratory of Forage & Herbivore, Chongqing Engineering Research Center for Herbivores Resource Protection and Utilization, Chongqing, 400715 People’s Republic of China; 2 Animal and Fish Production Department, Faculty of Agriculture (Alshatby), Alexandria University, Alexandria City, 11865 Egypt; 3 Present Address: Southwest University, Beibei, Chongqing, 400716 China

**Keywords:** DNA sequencing, Farm animals, IGFBP-3, RFLP

## Abstract

**Objective:**

A total of 205 animals from four Egyptian livestock species; cattle (n = 18), buffaloes (n = 12), sheep (n = 150) and goats (n = 25) were used in this study to detect polymorphism and perform comparative analysis for IGFBP-3 gene using DNA sequencing and (PCR–RFLP).

**Results:**

The amplified fragments were found to be of length 654 bp in sheep, 651 bp in cattle and 655 bp in buffalo. For Falahy goats, PCR was performed to amplify a 316 bp fragment from exon 2 of the IGFBP-3 gene. The digestion of 654 bp with *HaeIII* restriction enzyme yielded a single restriction pattern for goats, while for cattle, 3 genotypes were identified; (AA), (AB), and (BB). Moreover, for buffalo one genotype (AA) only was found with *HaeIII* and *TaqI* restriction enzymes, separately. Also, the digestion profile for goats with *HaeIII* revealed one pattern only. Nucleotide sequencing of the amplified fragments of IGFBP-3 gene in sheep, cattle, buffalo, and goat was submitted to the NCBI GenBank (Accession no. MG738671.1, MG738673.1, MG738674.1, and MG738672.1, respectively). The nucleotide sequencing analysis indicated similarity percentages in IGFBP-3 gene fragments of 88.54, 89.63 and 95.06% between “sheep and cattle”, “sheep and buffalo”, and “cattle and buffalo”, respectively.

## Introduction

There are inherent limitations in genome analysis of farm animal species in Egypt. Not enough genetic investigations on these animals are available, whether at the level of genes, QTL, or whole genome. Therefore, we tried through this modest attempt to open a window for studying the genome of these species, starting with some genes that have a major effect on some economic traits such as IGFBP-3. To the best of our knowledge, no studies are, yet, available on the comparisons in IGFBP-3 gene among domestic sheep, goat, cattle and buffalo species of Egypt [1, 2]. Additionally, the low heritability estimates and the subsequent slow genetic improvement via traditional selection approaches enhanced adopting molecular genetic techniques to achieve possible improvements through discovering candidate genes that have significant influences on such traits [2, 3].

Insulin-like growth factor binding proteins (IGFBPs) belongs to a family of at least six homologous proteins that bind IGFs and modulate many of their important biological actions [4]. It is recommended to be used as a marker for some body functions such as growth, body weight, reproduction, immunity, metabolism, and energy balance [8]. IGFBP-3 gene is responsible for the multiple and necessary effects of (IGF) [3–5].

Egyptian breeds from different species require the determination and characterization of several candidate genes responsible for the alteration of the economic traits behaviour. The objective of this study was to detect polymorphism in IGFBP-3 gene in Egyptian sheep, Falahy goats, Egyptian cattle and El-Beheiry buffaloes using a comparative sequence analysis (DNA sequencing) and PCR–RFLP techniques on samples of livestock species.

## Main text

### Materials and methods

#### Animals

Samples from four Egyptian farm animal species, namely cattle (n = 18; Egyptian cattle), buffaloes (n = 12; El-Beheiry breed), sheep (n = 150; 50 Rahmani, 50 Barki, and 50 Rahmani X Barki crosses) and goats (n = 25; Falahi breed) were obtained from four different geographical regions at the northern coast of Egypt, namely; Baltim farm (GPS: 31.579900, 31.174533)—Al Burlos (GPS: 31.580019, 31.174490)—Alexandria University ‘experimental station’ (GPS: 31.206208, 29.919704) and Matrouh farm (GPS: 31.336924, 27.205762). Blood samples of 5 ml each were collected from the jugular vein, using venojects, treated with 0.5 ml of 2.7% EDTA (Spark, UK) as an anticoagulant, kept in an icebox and transferred immediately to the lab. Sampled animals were apparently unrelated and their general characteristics correspond to the respective breeds conformation.

#### DNA extraction and amplification

Genomic DNA was extracted from blood samples with QIAGEN (QIAGEN GmbH, Hilden Germany) according to the manufacturer’s instructions. The isolated DNAs were separated by electrophoresis on 0.8% agarose (Bioshop, Germany) in 0.5 X TBE buffer prepared according to Sambrook et al. [6] and contained 0.5 μg/ml ethidium bromide (Sigma, Germany). The electrophoresis run was performed using apparatus with power supply (Biometra, USA) and visualized by UV trans-illuminator and Gel documentation system (Gel Doc.Alpha-chem.Imager, USA).

For the sampled Egyptian sheep, cattle, and buffaloes, a region of IGFBP-3 gene spanning over a part of exon 2, complete intron 2, exon 3 and a part of intron 3 was amplified using primer AASN-P1; (F: 5-CCAAGC GTG AGA CAG AAT AC-3),(R:5-AGG AGG GAT AGG AGC AAG AT-3) [3–8]. PCR for Falahy goats was performed to amplify a 316 bp fragment from exon 2 of the IGFBP-3 gene using a primer as described by Liu et al. [9], AASN-P2; (F:5′-GAA ATG GCA GTG AGT CGG-3′), (R:5′-TGG GCT CTT GAG TAA TGG TG-3′).

The amplification was performed using (iQ SYBR Green Supermix, USA), 10 p.mol of each primer and 80–100 ng of genomic DNA were processed under the following amplification conditions: 94 °C/5 min, followed by 35 cycles of 94 °C/1 min, 60 °C/1 min, 72 °C/1 min and a final extension step at 72 °C/2 min for (AASN-P1) primer. As for (AASN-P2) primer, the conditions were 94 °C/5 min, followed by 34 cycles of 94 °C/1 min, 63 °C/1 min, 72 °C/1 min and a final extension step at 72 °C/2 min. The amplification was carried out using a DNA Thermo-cycler Gene Amp 6700 (Applied Bio-system, USA).

#### Nucleotide sequence analysis

Automated DNA sequence analysis was carried out on both strands by the DNA sequencing service lab of the Korean Research Institute of Bioscience and Biotechnology with an ABI Prism 3100 apparatus. Database similarity searches were performed with the NCBI/BLAST/blast network service at the National Center for Biotechnology Information (NCBI) (http://www.ncbi.nlm.nih.gov). The resulted sequences were analyzed using MEGA 6 v.4, Finch T.V 1.01, and Blast 2.0 software to detect Single Nucleotide Polymorphism (SNPs) between sequences. The sequences were deposited in GenBank (Accession Numbers: MG738673.1, MG738674.1, MG738671.1 and MG738672.1 for cattle, buffalo, sheep, and goat, respectively). Analysis of translated protein of IGFBP-3 gene sequences of tested animals were generated by ExPASy program (http://web.expasy.org/translate).

#### Restriction fragment length polymorphism (RFLP)

The RFLP was used to detect genotyping differences between and within sheep groups, cattle, buffalo, and goat using the PCR of target genes. The PCR amplicons of the IGFBP-3 gene were digested with *HaeIII* for all tested animals, also *TaqI* was used separately for buffalo only beside *HaeIII* (Jena Bioscience, Germany). Defining restrictions sites before digestion with restriction enzymes was achieved by the NEB cutter program (http://www.labtools.us/nebcutter-v2-0) [10]. The RFLP-PCR reaction volume was 25 μl, consisted of 11–12 μl H_2_O, 2 μl 10X digestion buffer, 5–10 units restriction enzyme (5 unit/1 μl) in addition to 10 μl amplified DNA. All reactions were incubated at 37 °C for *HaeIII* and at 65 °C for *TaqI* for 16 h. Twenty microliter of each reaction were separated by electrophoresis on 3% agarose gel and visualized by UV trans-illuminator and gel documentation system (Gel Doc. Alpha-chem. Imager, USA).

### Results and discussion

#### Amplification, manipulation, and digestion

**Fig. 1:**
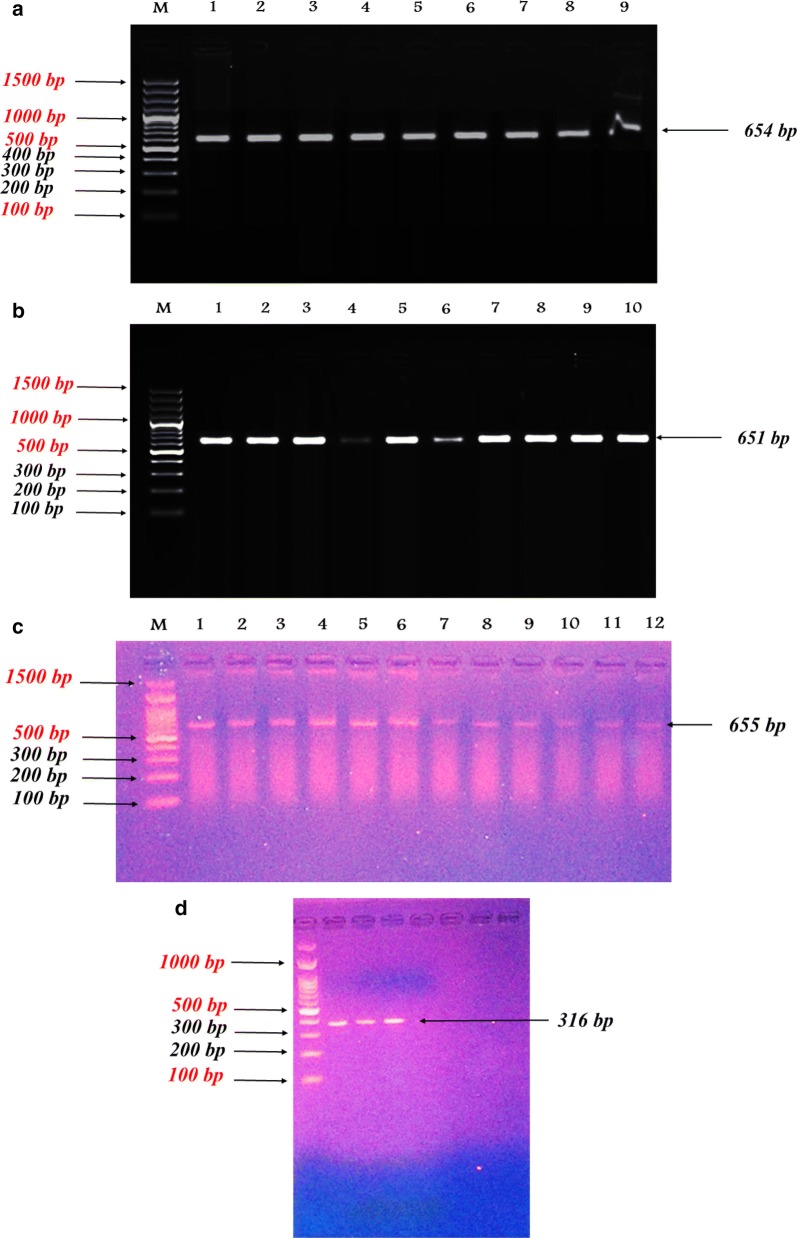
PCR amplification of IGFBP-3 gene for sheep (**a**), Cattle (**b**), Buffalo (**c**) and goat (**d**). M;100 bp DNA ladder

**Fig. 2:**
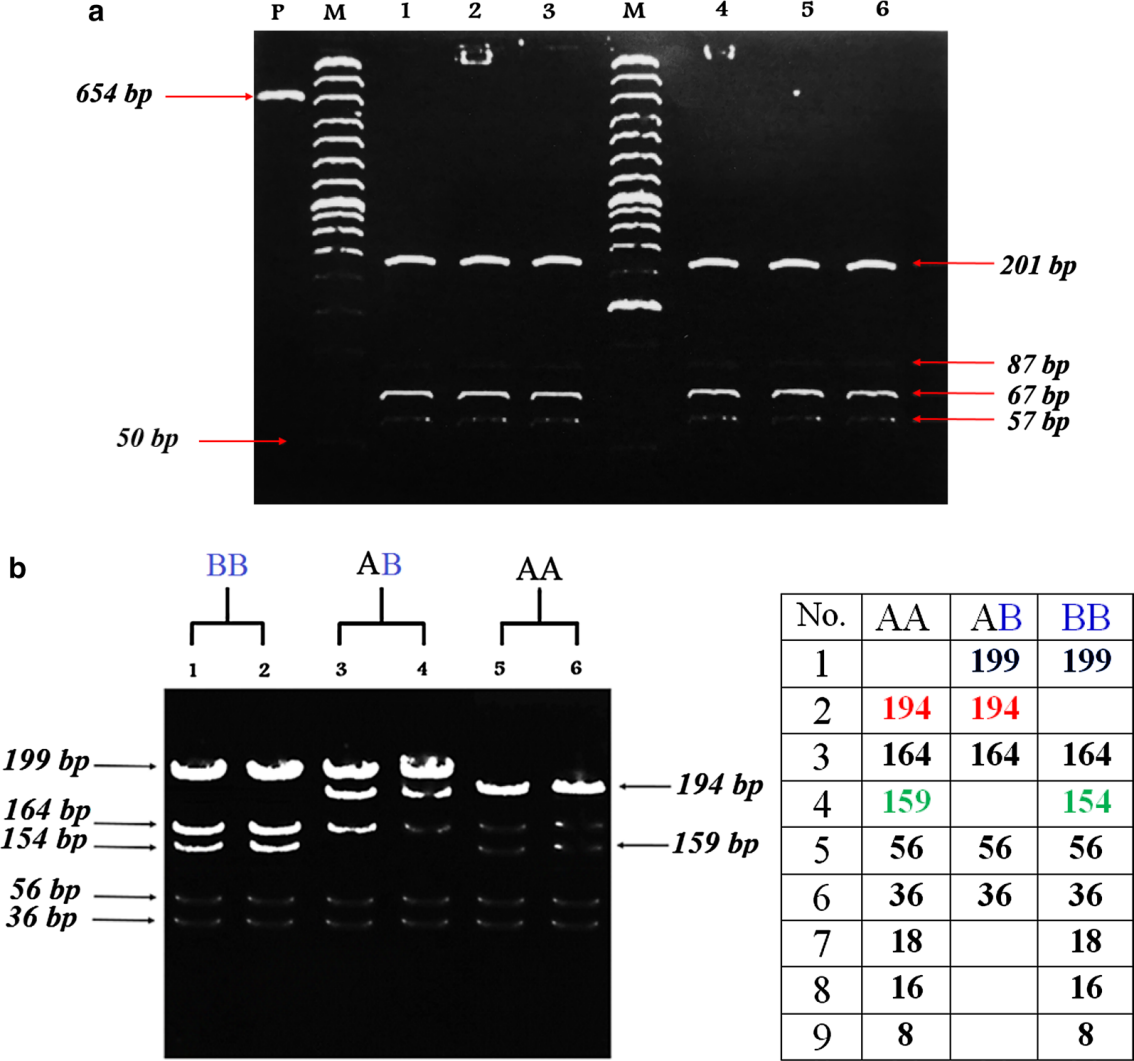
**a** The PCR products of the IGFBP-3 gene from genomic DNA of tested breeds digested by *HaeIII*. Lanes: 1 and 2; Rahmani, lanes: 3 and 4; Barki, and lanes: 5 and 6; Rahmani X Barki crosses. M, 50 bp DNA ladder, P; PCR product for IGFBP-3. The digestion with *HaeIII* revealing a single pattern only for 8 DNA fragments sized 201, 201, 87, 67, 57, 18, 16 and 7 bp and indicating absence of a polymorphism in tested sheep breeds for IGFBP-3 gene. The restriction fragments with sizes; 18, 16 and 7 bp were not seen on the gel. **b** *HaeIII* restriction pattern of cattle IGFBP-3 gene. Lane M: 100-bp ladder marker. Lanes 1 and 2; homozygous (BB) genotype with 8 restricted fragments at 199, 164, 154, 56, 36, 18, 16 and 8 bp, Lanes 3 and 4; heterozygous (AB) genotype with 5 restricted fragments at 199, 194, 164, 56 and 36 bp and Lanes 5 and 6; homozygous (AA) genotype with 8 restricted fragments at 194, 164, 159, 56, 36, 18, 16 and 8 bp

This research note concerns mainly the differentiation between and within different livestock species for IGFBP-3 gene. It also spotlights the association between polymorphism of IGFBP-3 and the performance of different species of farm animals for some economical traits. PCR amplification for the tested animals produced an amplified 654 bp fragment for Egyptian sheep IGFBP-3 gene comprised of part of exon 2, complete intron 2, exon 3 and a part of intron 3 (Fig. 1a), an amplified 651 bp fragment for Egyptian cattle (Fig. 1b) and an amplified 655 bp fragment for El-Beheiry buffalo (Fig. 1c), while the amplified 316 bp fragment for Falahi goat IGFBP-3 gene was comprised of part of exon 2 (Fig. 1d). The PCR products of IGFBP-3 gene obtained for sheep after digestion with *HaeIII* (Fig. 2a) showed a digestion profile revealing only one pattern for eight DNA fragments sized 201, 201, 87, 67, 57, 18, 16 and 7 bp and indicated absence of polymorphism in sheep IGFBP-3 gene. The restriction fragments with sizes; 18, 16 and 7 bp were not seen on the gel.

The present results agree with those of Kumar et al. [3] who studied the genetic diversity among the Indian breeds of sheep; Marwari, Mandya, Madras, Red Muzaffarnagari and Banur based on sequencing and digestion profile of IGFBP-3 gene with *HaeIII* and reported that; the digestion profile revealed only one pattern with eight DNA fragments sized 201, 201, 87, 67, 56, 19, 16 and 7 bp for the tested animals and, consequently, no polymorphism was detected. Also, Choudhary et al. [11] reported that; all sheep possess intact *HaeIII* restriction site (GG↓CC) at the base number 300 of IGFBP-3 gene sequence indicating also, an absence of polymorphism at this site.

For Egyptian cattle, three genotypes were identified: (1) Lanes 1 and 2; homozygous (BB) genotype with 8 restricted fragments at 199, 164, 154, 56, 36, 18, 16 and 8 bp, (2) Lanes 3 and 4; heterozygous (AB) genotype with 5 restricted fragments at 199, 194, 164, 56 and 36 bp and (3) Lanes 5 and 6; homozygous (AA) genotype with 8 restricted fragments at 194, 164, 159, 56, 36, 18, 16 and 8 bp (Fig. 2b). The restriction fragments with sizes; 18, 16 and 8 bp were not seen on the gel. The polymorphism in cattle was due to C→A (GG↓CC to GG AC) transition/mutation in exon (3) of the gene at the 451st base position of sequence for allele (A), while was due to (GG↓CC to AG AC) transition/mutation in exon (3) of the gene at the 456th base position of cattle sequence for allele (B), (Additional file 1: Figure S3). These results were in agreement with those of Shukla [12]; Kumar et al. [3], and Choudhary [11] who detected three genotypes identified in Jersey and exotic Holstein–Friesian cattle. Moreover, Othman et al. [13] found that; the digestion of PCR products with the restriction enzyme of *HaeIII* revealed three genotypes in some local cattle;(AA), (CC), and (AC) with frequencies nearly 22%, 22%, and 56%, respectively.

As for the buffalo, the presence of *TaqI* site characterized by a single homozygous genotype possessing two fragments of sizes 415 and 240 bp were observed (Additional file 1: Figure S1A). As for *HaeIII* restriction enzyme, all screened buffaloes showed only one genotype (AA) with restriction fragments of sizes 201, 165, 154, 56, 36, 19, 16 and 8 bp (Additional file 1: Figure S1B) in accordance with those reported by Choudhary [11] on six breeds of buffalo, though the sizes of restriction fragments were different (201, 165, 154, 56, 36, 19, 16 and 8 bp). The above results mean the lack of detected polymorphism among studied buffalo breeds with respect to IGFBP-3 gene. Similarly, results of Padma et al. [14] on 157 Indian Murrah, Surti, Jaffarabadi and Nagpuri riverine buffaloes, revealed that; the digestion with *HaeIII* yielded single restriction pattern of 8 fragments of sizes 201, 165, 154, 56, 36, 19, 16 and 8 bp for all animals and with *TaqI* and *MspI* also produced single restriction pattern yielding fragments of sizes 240, 415 bp and 145, 510 bp, respectively. This again shows the non-polymorphic nature of restriction sites in buffalo.

Finally, the PCR profiles of tested goats IGFBP-3 gene digested with *HaeIII* revealed one pattern only for three DNA fragments sized 263, 58 and 8 bp (Additional file 1: Figure S2), the restriction fragment with size 8 bp was not seen on the gel. However, the study of Lan et al. [5] on goats detected polymorphisms in the IGFBP-3 gene by PCR-SSCP and DNA sequencing methods. Though the associations of *HaeIII* and *XspI* PCR-RFLPs for goat IGFBP-3 locus with milk traits were analyzed, no significant statistical results were found.

Additional file 1: Figure S3 shows the diagrammatic representation of exon–intron regions of the tested animals of different species and restriction enzymes sites (*HaeIII* and *TaqI*) of the amplified IGFBP-3 gene fragments.

#### Nucleotide sequence comparison

Nucleotide sequencing of the amplified fragments of the IGFBP-3 gene of sheep, goats, cattle, and buffaloes were submitted to the NCBI GenBank (Accession no. MG738671.1, MG738672.1, MG738673.1 and MG738674.1, respectively) (Additional file 1: Figure S4). The nucleotide sequence analysis performed by Blastn (https://blast.ncbi.nlm.nih.gov/Blast) indicated that the similarity percentages of IGFBP-3 gene fragment between (sheep and cattle was 88.54%), between (sheep and buffalo was 89.63%), while between (cattle and buffalo was 95.06%) (Additional file 1: Figure S5).

#### Protein sequence comparison

The partial part of exon 2, complete intron 2, exon 3 and a part of intron 3 present in the sequence of the amplified IGFPB-3 gene fragments of tested animals as generated by ExPASy program (http://web.expasy.org/translate) and the comparison of amino acids obtained by MEGA-6 VERSION 4 are in (Additional file 1: Figure S6). The protein sequence of sheep is different from that of cattle and buffalo by 18 amino acids. The display of amino acids (Table 1) accounted for approximately 70% similarity in sequence between sheep groups and bovine species vs. cattle and buffalo. However, the study of Kumar et al. [3] indicated approximately 93% similarity in the amino acid sequence for sheep with cattle and buffalo.

**Table 1:** The different amino acids in sheep as compared with cattle and buffalo, which were obtained from a part of exon 2, complete exon 3 for each species

No.	1	2	3	4	5	6	7	8	10
Sheep	D Aspartic	S Serine	Q Glutamine	Q Glutamine	L Leucine	L Leucine	Q Glutamine	T Threonine	K Lysine
Cattle and Buffalo	T Threonine	Q Glutamine	S Serine	S Serine	Y Tyrosine	S Serine	S Serine	G Glycine	S Serine

No.	10	11	12	13	14	15	16	17	18
Sheep	Q Glutamine	L Leucine	A Alanine	C Cysteine	L Leucine	R Arginine	F Phenylalanine	L Leucine	Q Glutamine
Cattle and Buffalo	S Serine	C Cysteine	P Proline	Y Tyrosine	R Arginine	S Serine	S Serine	R Arginine	P Proline

## Limitations

More research on IGFBP-3 gene of Egyptian endogenous livestock species is required for detection of polymorphism, comparison of gene sequencing and tracing the evolutionary relatedness for the gene sight among groups and breeds of farm animals within and between species. Relating the obtained results to the performance of the animals for the important economic traits will help to adapt applicable combined traditional and molecular selection programmes and building a link between the gene differentiation and performance. The practice of increasing the sample size is necessary to move the limitations of random drift.

## Supplementary information

**Additional file 1: Figure S1. (A)** - Digestion pattern of PCR amplification of IGFBP-3 gene from the genomic DNA of tested buffalo breeds with *Taq*-*I* revealing a single homozygous genotype and a non-polymorphic as two fragments of sizes 415 and 240 bp were observed, M; 50 bp DNA ladder. **(B) -** A 655 bp sequence of IGFBP-3 gene of Egyptian buffalo (*NCBI accession no. MG738674*) in this study with restriction sites for the *HaeIII* restriction enzyme, thus all screened buffaloes showed only one genotype (AA) with restriction fragments of sizes 201, 165, 154, 56, 36, 19, 16 and 8 bp. **Figure S2.** Digestion pattern of PCR amplification of the IGFBP-3 gene from the genomic DNA of tested goat breeds, the digested with *HaeIII* revealed one pattern only for three DNA fragments sized 263, 58 and 8 bp, the restriction fragment with size 8 bp was not seen on the gel, M; 50 bp DNA ladder. **Figure S3.** The diagrammatic representation of exon–intron regions of animals tested and restriction enzymes sites (*HaeIII* and *TaqI*) of amplified IGFBP-3 gene fragment in sheep, cattle, buffalo, and goat. **Figure S4.** (A) - A 654 bp sequence of the IGFBP-3 gene for Egyptian sheep (*NCBI accession no. MG738671.1*). (B)- A 651 bp sequence of IGFBP-3 gene for Egyptian Cattle (*NCBI accession no. MG738673.1*). (C) - A 655 bp sequence of IGFBP-3 gene for Egyptian buffalo (*NCBI accession no. MG738674.1*). (D) - A 316 bp sequence of IGFBP-3 gene for Egyptian goat (*NCBI accession no. MG738672.1*) in the current study. **Figure S5.** Nucleotide sequence comparison of amplified IGFBP-3 gene of sheep, cattle and buffalo using (MEGA-6) Molecular Evolutionary Genetics Analysis, VERSION 4 (http://en.bio-soft.net/tree/MEGA.html). **Figure S6.** Comparative analysis of protein sequence of IGFBP-3 gene of sheep, cattle and buffalo using ExPASy program (http://web.expasy.org/translate) and the comparison of amino acid using (MEGA-6), VERSION 4 (http://en.bio-soft.net/tree/MEGA.html).

## Data Availability

All data generated or analyzed during this study are included in this manuscript and its additional information files.

## Abbreviations

IGFBP-3: insulin-like growth factor binding proteins-3
PCR: polymerase chain reaction
RFLP: restriction fragment length polymorphism
QTL: quantitative trait loci
IGF: insulin-like growth factor
