# Supplementary material for: Comparative analysis of IGFBP-3 gene sequence in Egyptian sheep, cattle, and buffalo
Source: BMC Res Notes. 2019 Sep 23;12:623. doi: 10.1186/s13104-019-4657-6 (PMC6757409; doi:10.1186/s13104-019-4657-6)
Supplement: Supplementary file 1 — Additional file 1: Figure S1. (A) - Digestion pattern of PCR amplification of IGFBP-3 gene from the genomic DNA of tested buffalo breeds with Taq-I revealing a single homozygous genotype and a non-polymorphic as two fragments of sizes 415 and 240 bp were observed, M; 50 bp DNA ladder. (B) - A 655 bp sequence of IGFBP-3 gene of Egyptian buffalo (NCBI accession no. MG738674) in this study with restriction sites for the HaeIII restriction enzyme, thus all screened buffaloes showed only one genotype (AA) with restriction fragments of sizes 201, 165, 154, 56, 36, 19, 16 and 8 bp. Figure S2. Digestion pattern of PCR amplification of the IGFBP-3 gene from the genomic DNA of tested goat breeds, the digested with HaeIII revealed one pattern only for three DNA fragments sized 263, 58 and 8 bp, the restriction fragment with size 8 bp was not seen on the gel, M; 50 bp DNA ladder. Figure S3. The diagrammatic representation of exon–intron regions of animals tested and restriction enzymes sites (HaeIII and TaqI) of amplified IGFBP-3 gene fragment in sheep, cattle, buffalo, and goat. Figure S4. (A) - A 654 bp sequence of the IGFBP-3 gene for Egyptian sheep (NCBI accession no. MG738671.1). (B)- A 651 bp sequence of IGFBP-3 gene for Egyptian Cattle (NCBI accession no. MG738673.1). (C) - A 655 bp sequence of IGFBP-3 gene for Egyptian buffalo (NCBI accession no. MG738674.1). (D) - A 316 bp sequence of IGFBP-3 gene for Egyptian goat (NCBI accession no. MG738672.1) in the current study. Figure S5. Nucleotide sequence comparison of amplified IGFBP-3 gene of sheep, cattle and buffalo using (MEGA-6) Molecular Evolutionary Genetics Analysis, VERSION 4 (http://en.bio-soft.net/tree/MEGA.html). Figure S6. Comparative analysis of protein sequence of IGFBP-3 gene of sheep, cattle and buffalo using ExPASy program (http://web.expasy.org/translate) and the comparison of amino acid using (MEGA-6), VERSION 4 (http://en.bio-soft.net/tree/MEGA.html). [file 13104_2019_4657_MOESM1_ESM.docx]

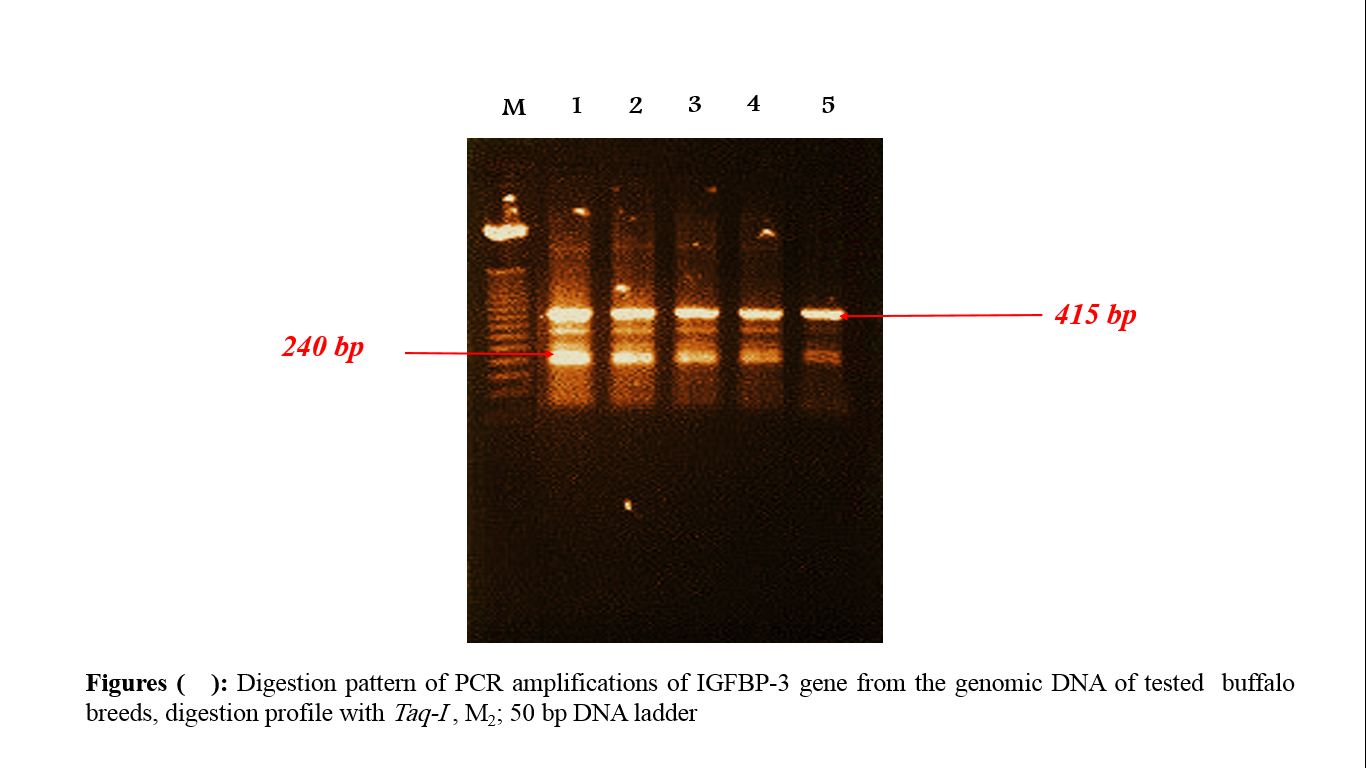
**(A)**

**(B)**

| TCTTGTGGATGTGGGGGTGGGG^CCACCTGG^CCCTGGGTATCCAGAGATCACAGGGTCACCATTACTCAAGAGCCCAGCAGTTACTCCAGTGGTCCTGCTGATGCACTGAGCAGCTGTGAGCCCCTGCTTACAGAAGGGATATTGACCCTCCCCTATGGCAGAGATCCCAGGAGAATCAGTGCACTGCTCTCCAGG^CCTCGGCTGGGCAGAGCAGTGTTCTCACAAAGCTGG^CCTCTTTTTGTTCACTTGG^CCTCTGAGTGTCCTGG^CCTGTGTATCCCTGTCCCAGTCCTGTAGCTTGCCCTGGGGAATCACAAGAGAGACAGGGGGCTGTGGTTGGCATCTGCACAGGAACAGTGACAACTAAATCAGACAAAAGATACTCGAGGAGCACGTGGTCAGTCCCCTGGGTGTTACAGGGTTTTATCAGACACAGAGTTCCCAGGTAACCCATGCCTCCTTCCCAGGGG^CCCTGCCGCCGGGAAATGGAAGACACGCTGAACCACCTGAAGTTCCTGAACATGCTCAGCCCCAGGGGCATCCACATTCCCAACTGCGACAAGAAGGGCTTCTACAAGAAAAAGCAGGTGCCCTCCTGG^CCTGTGTATCCCTGTAACAGTCCTGTAGGTTGCCCAGAAATC |
| --- |

**Figure S1. (A)** - Digestion pattern of PCR amplification of IGFBP-3 gene from the genomic DNA of tested buffalo breeds with *Taq-I* revealing a single homozygous genotype and a non-polymorphic as two fragments of sizes 415 and 240 bp were observed, M; 50 bp DNA ladder. **(B) -** A 655 bp sequence of IGFBP-3 gene of Egyptian buffalo (*NCBI accession no. MG738674*) in this study with restriction sites for the *HaeIII* restriction enzyme, thus all screened buffaloes showed only one genotype (AA) with restriction fragments of sizes 201, 165, 154, 56, 36, 19, 16 and 8 bp.

**
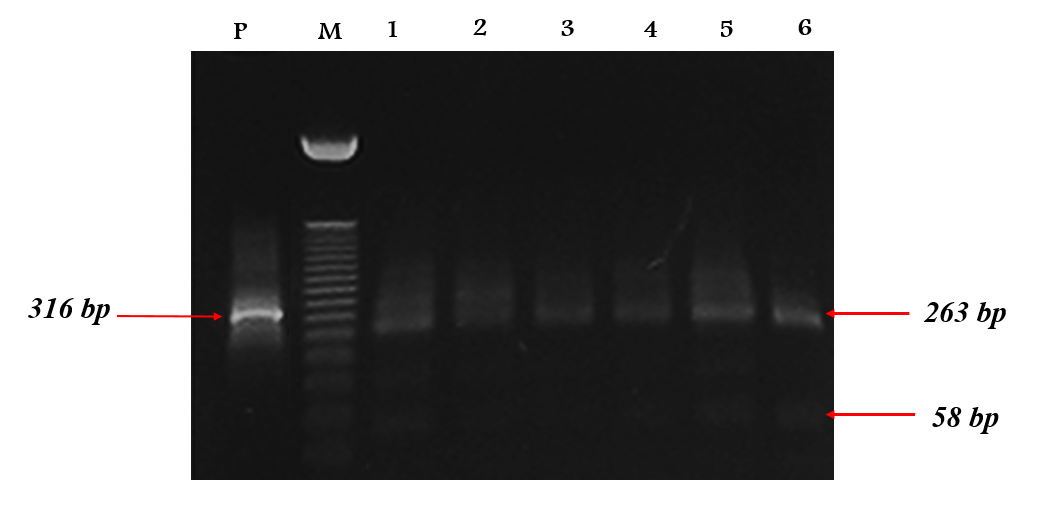
**

**Figure S2.** Digestion pattern of PCR amplification of the IGFBP-3 gene from the genomic DNA of tested goat breeds, the digested with *HaeIII* revealed one pattern only for three DNA fragments sized 263, 58 and 8 bp, the restriction fragment with size 8 bp was not seen on the gel, M; 50 bp DNA ladder.


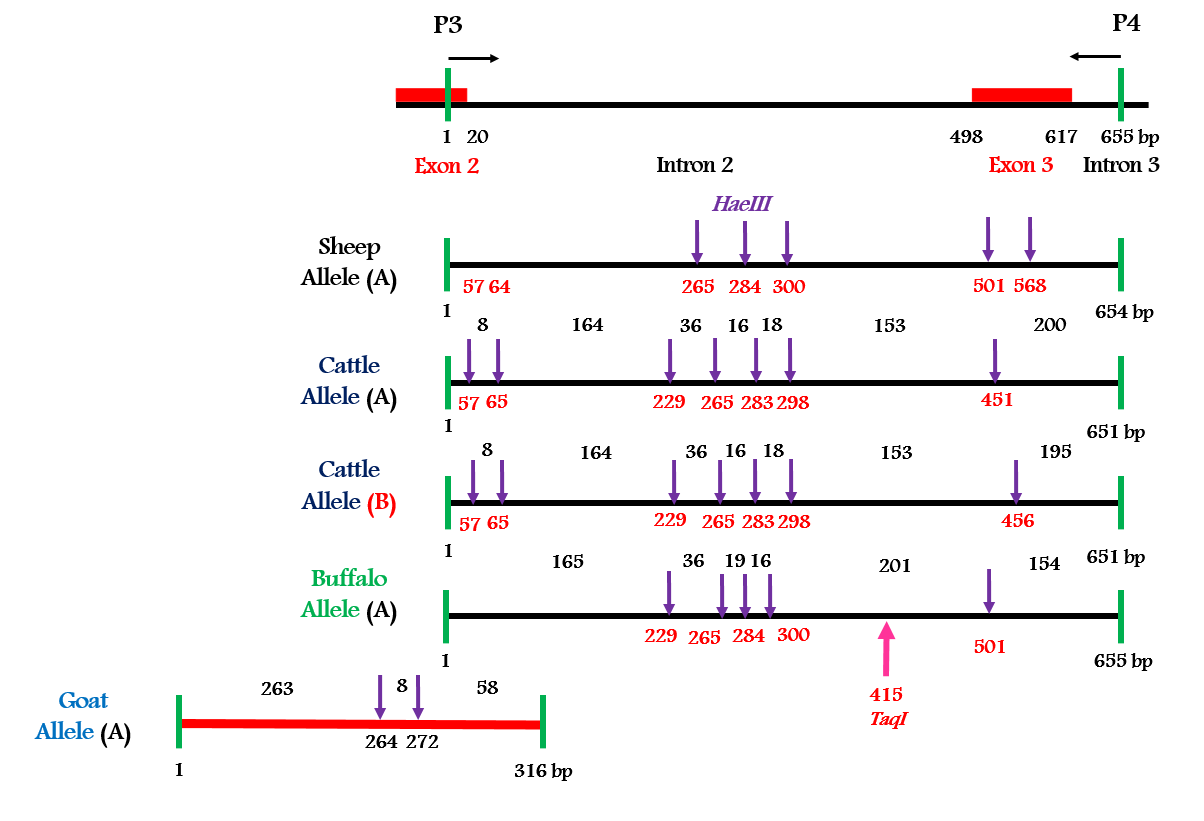


**Figure S3.** The diagrammatic representation of exon-intron regions of animals tested and restriction enzymes sites (*HaeIII* and *TaqI*) of ampliﬁed IGFBP-3 gene fragment in sheep, cattle, buffalo, and goat.


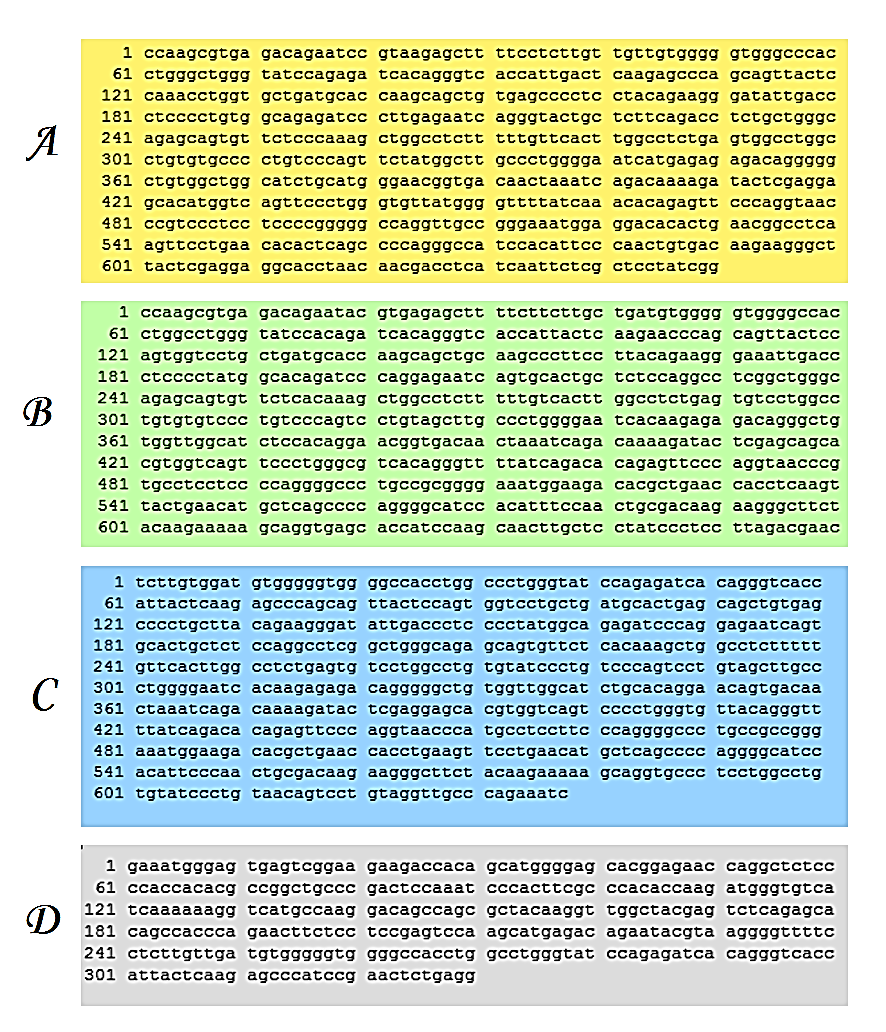


**Figure S4.** (A) - A 654 bp sequence of the IGFBP-3 gene for Egyptian sheep (*NCBI accession no. MG738671.1*). (B)- A 651 bp sequence of IGFBP-3 gene for Egyptian Cattle (*NCBI accession no. MG738673.1*). (C) - A 655 bp sequence of IGFBP-3 gene for Egyptian buffalo (*NCBI accession no. MG738674.1*). (D) - A 316 bp sequence of IGFBP-3 gene for Egyptian goat (*NCBI accession no. MG738672.1*) in the current study.


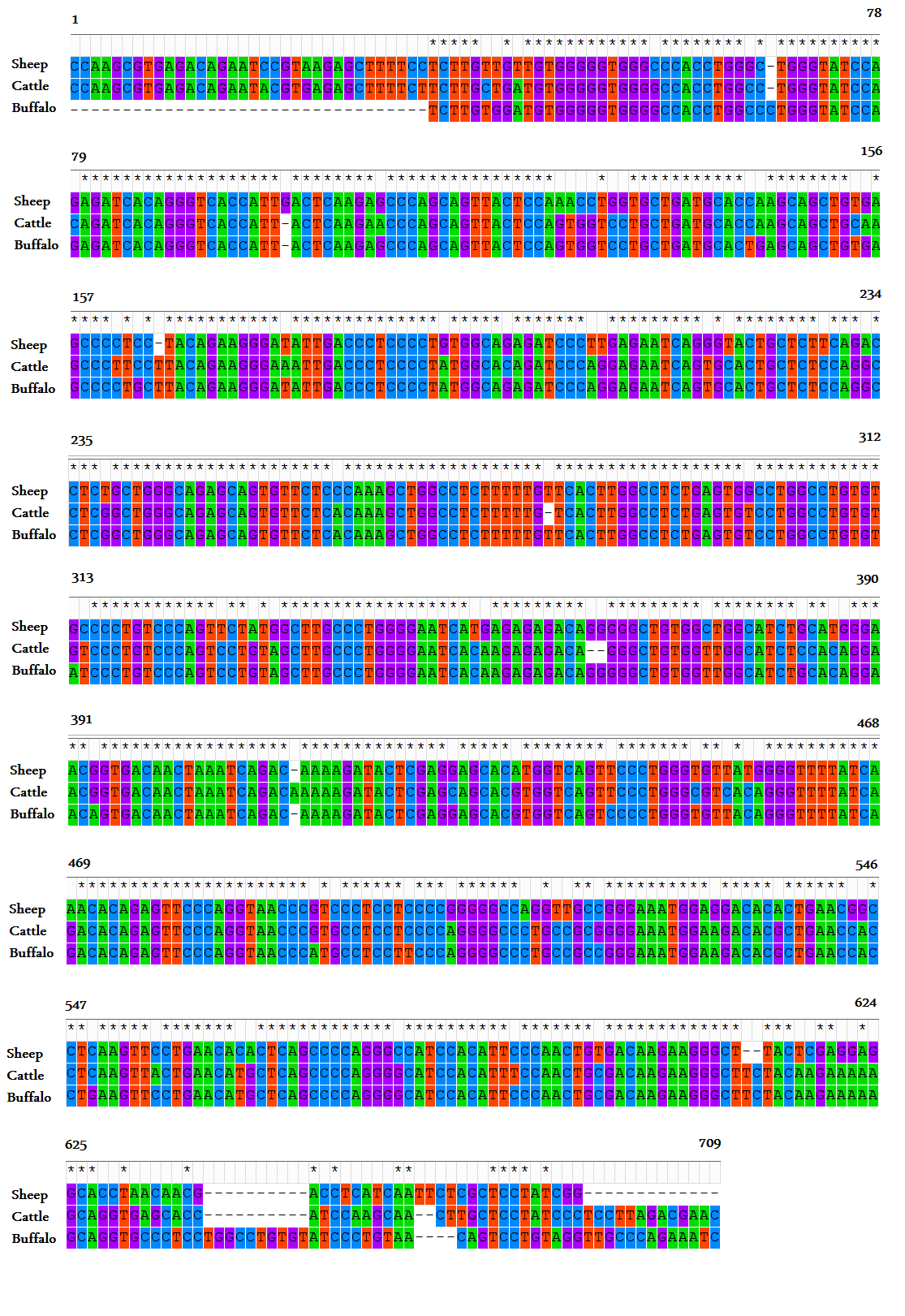


**Figure S5.** Nucleotide sequence comparison of ampliﬁed IGFBP-3 gene of sheep, cattle and buffalo using (MEGA-6) Molecular Evolutionary Genetics Analysis, VERSION 4 (<http://en.bio-soft.net/tree/MEGA.html>)


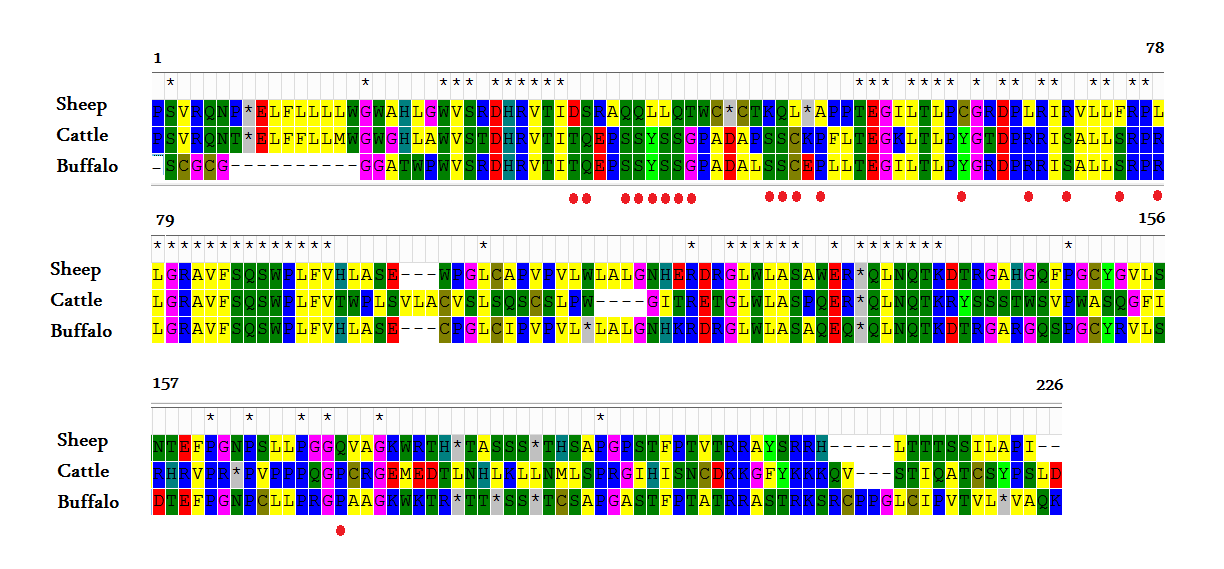


**Figure S6.** Comparative analysis of protein sequence of IGFBP-3 gene of sheep, cattle and buffalo using ExPASy program (<http://web.expasy.org/translate>) and the comparison of amino acid using (MEGA-6), VERSION 4 (http://en.bio-soft.net/tree/MEGA.html).
